# World Association of HPB Surgery: Third World Congress of HPB Surgery

**DOI:** 10.1155/1990/26027

**Published:** 1990

**Authors:** Robin Williamson

**Affiliations:** Department of Surgery Royal Postgraduate Medical School Hammersmith Hospital Du Cane Road London W12 0NN United Kingdom


					HPB Surgery, 1990, Vol. 2, p. 303
Reprints available directly from the publisher
Photocopying permitted by license only
1990 Harwood Academic Publishers GmbH
Printed in the United Kingdom
World Association of HPB Surgery
THIRD WORLD CONGRESS OF HPB SURGERY
London, U.K. 3-8 June 1990
The Third World Congress of HPB Surgery was held at Kensington Town Hall,
London, from 3-8 June. There were about .850 participants from 60 different
countries of the world. Proceedings were started by the Band of the Grenadier
Guards at a Welcome Reception on the Sunday evening. There followed four and a
half days of scientific programme, including some 200 free papers, 250 posters and
40 films or videos. Eight eminent experts delivered Keynote Addresses and another
eight Update Lectures. A feature of the meeting was three interactive case
presentations, in which the audience could vote on the management of a particular
clinical case presented by a professor. There was a Government Reception on the
Monday night at the Banqueting House, Whitehall and the Congress Banquet was
held on the Wednesday night at The Brewery, City of London.
Dr Martin Adson (USA) completed his term of office as President of the World
Association of HPB Surgery during the meeting, and the incomeing President is
Professor Niels van der Heyde (Netherlands). Other new Officers were elected as
follows: President-Elect, S. Bengmark (Sweden); Vice-President, A.K.C. Li
(Hong Kong); Treasurer, B. Langer (Canada); Secretary-General, R.C.N.
Williamson (UK); Chairman of the Scientific Committee, H.A. Pitt (USA). These
six Officers and the immediate Past President (Dr Adson) will now serve on the
Executive Committee from 1990-92, together with seven Members-at-Large
elected as follows: G. Gozzetti (Italy), E. Moreno-Gonzalez (Spain), K.E.F.
Hobbs (UK), D. Houssin (France), B. Kekis (Greece, R. Strong (Australia), T.
Tobe (Japan).
A new Scientific Committee was elected under the chairmanship of Dr Pitt as
follows: I.S. Benjamin (UK), S.C.S. Cheung (Hong Kong), D. Franco (France),
B.W. Jeppsson (Sweden), C-G Ker (Taiwan), W-Y. Lau (Hong Kong), J. Scheele
(FRG), S. Strasberg (Canada), T. Suzuki (Japan), O. T. Terpstra (Netherlands), J.
Toouli (Australia).
The Fourth World Congress of HPB Surgery will be held in Hong Kong in June
1992, with Professor Arthur Li as Chairman of the Local Organising Committee,
and the Fifth World Congress in Boston, USA in 1994.
Robin Williamson
Secretary-General, Department of Surgery
Royal Postgraduate Medical School
Hammersmith Hospital, Du Cane Road
London W12 0NN, United Kingdom
303

				

